# The Antidepressant Action of Fluoxetine Involves the Inhibition of *Dlx5/6* in Cortical GABAergic Neurons through a TrkB-Dependent Pathway

**DOI:** 10.3390/cells13151262

**Published:** 2024-07-26

**Authors:** Rym Aouci, Anastasia Fontaine, Amïn Vion, Lou Belz, Giovanni Levi, Nicolas Narboux-Nême

**Affiliations:** Molecular Physiology and Adaption, UMR7221 CNRS, Museum National d’Histoire Naturelle, 75005 Paris, France; rym.aouci@mnhn.fr (R.A.); anastasia.fontaine@mnhn.fr (A.F.); lou.belz@mnhn.fr (L.B.); glevi@mnhn.fr (G.L.)

**Keywords:** fluoxetine, *Dlx5/6*, TrkB-CREB, Parvalbumine, depression

## Abstract

Major depressive disorder (MDD) is a complex and devastating illness that affects people of all ages. Despite the large use of antidepressants in current medical practice, neither their mechanisms of action nor the aetiology of MDD are completely understood. Experimental evidence supports the involvement of Parvalbumin-positive GABAergic neurons (PV-neurons) in the pathogenesis of MDD. *DLX5* and *DLX6* (*DLX5/6*) encode two homeodomain transcription factors involved in cortical GABAergic differentiation and function. In the mouse, the level of expression of these genes is correlated with the cortical density of PV-neurons and with anxiety-like behaviours. The same genomic region generates the lncRNA *DLX6-AS1,* which, in humans, participates in the GABAergic regulatory module downregulated in schizophrenia and ASD. Here, we show that the expression levels of *Dlx5/6* in the adult mouse brain are correlated with the immobility time in the forced swim test, which is used to measure depressive-like behaviours. We show that the administration of the antidepressant fluoxetine (Flx) to normal mice induces, within 24 h, a rapid and stable reduction in *Dlx5*, *Dlx6* and *Dlx6-AS1* expression in the cerebral cortex through the activation of the TrkB-CREB pathway. Experimental *Dlx5* overexpression counteracts the antidepressant effects induced by Flx treatment. Our findings show that one of the short-term effects of Flx administration is the reduction in *Dlx5/6* expression in GABAergic neurons, which, in turn, has direct consequences on *PV* expression and on behavioural profiles. Variants in the *DLX5/6* regulatory network could be implicated in the predisposition to depression and in the variability of patients’ response to antidepressant treatment.

## 1. Introduction

Depression and anxiety disorders are common psychiatric conditions with high comorbidity and massive medical, social and financial burdens. The cellular and molecular substrate of these disorders, and the search for alleviating treatments, have been a matter of debate for decades. The “monoamine hypothesis”, which identifies a deficiency or imbalances in monoamine neurotransmitters, in particular serotonin, as the cause of depression constitutes the most accepted explanation for the origin of these diseases. The monoamine hypothesis is supported by the properties of many antidepressants that target the monoamine systems. In particular, “selective serotonin reuptake inhibitors” (SSRIs), the most prescribed class of antidepressants, block the membranous serotonin transporter (SERT, SLC6A4, 5-HTT), resulting in increased synaptic serotonin levels and in adaptive responses from the serotonergic system [1]. However, the aetiology of depression is not fully established, and considering the heterogeneity of symptoms, underlying genetics and treatment responses, it is generally assumed that the cause of MDD is likely multifactorial. A major limitation of the monoamine hypothesis is that it does not fully explain the kinetics of SSRI action. In particular, the behavioural benefits of fluoxetine (Flx), one of the commonly prescribed SSRIs, can be typically observed only after 3–4 weeks of chronic treatment, suggesting the involvement of a slow adaptive response, such as increased hippocampal adult neurogenesis [2,3].

The leading monoamine hypothesis of depression coexists with other theories [4]. Among them, the “GABAergic deficit hypothesis” for depression implicates GABAergic system defects in common phenotypes of MDD and suggests that antidepressant therapies act on GABAergic neurons [5,6].

This theory is supported by results from clinical studies that showed that depression implicates functional defects in cortical GABAergic inhibition [7] and can be associated with reduced GABA brain levels [8,9], reduced expression of glutamic acid decarboxylase, the limiting enzymes for GABA synthesis [10,11] or reduced density or function of GABAergic interneurons subclasses [11,12].

In addition to its action on the serotonin transporter, fluoxetine treatment induces several other changes in the brain [13]. For instance, Flx administration indeed affects the major class of GABAergic cortical neurons: Parvalbumin (PV)-positive interneurons, which are critical regulators of brain functions, including information processing, fear and stress-related behaviours [14,15,16].

Chronic Flx administration induces a strong reduction in *Parvalbumin* expression and a shrinkage of their extracellular matrix, the perineuronal net (PNN), a hallmark of these neurons’ maturity [17,18]. By these actions, fluoxetine treatment induces an adolescent-like period of brain plasticity that allows a strong response to environmental cues that might participate in the antidepressant effect of Flx by allowing a partial rewiring of the brain [17]. Interestingly, this “dematuration”, or “iPlasticity”, requires the direct binding of Flx to a dimerized TrkB receptor (neurotrophic receptor tyrosine kinase 2), independently from the serotonin transporter SERT [19,20]. Interestingly, the activation of TrkB signalling selectively in PV neurons is sufficient to promote iPlasticity, by inducing cellular and neuronal network alterations reminiscent of fluoxetine administration, facilitating the influence of environmental factors on neuronal networks [21].

Recent findings have shown that both typical (e.g., Flx) and fast-acting antidepressants such as ketamine directly bind to TrkB, suggesting that TrkB is a direct, acute and common target for several classes of antidepressants. It appears, therefore, that direct activation of TrkB is a critical step to permit the rapid biological action of antidepressants [22]. Other studies have shown that antidepressants also promote the synthesis and release of the TrkB endogenous ligand BDNF (brain-derived neurotrophic factor) [23,24,25]. Together, these experiments suggest an important role of TrkB signalling modulation for the cellular and behavioural effects of antidepressants [24,26]. This role is independent from the serotonin transporter SERT [19] and, therefore, from variations in serotonin brain concentrations.

Among the downstream effectors of TrkB, the CREB pathway (cAMP response element-binding protein) appears to be involved in antidepression [27,28]. CREB expression and transcription functions have been shown to be increased after fluoxetine treatment, independently from SERT [19,23]. Another intracellular effector of TrkB, the mTOR pathway (mechanistic target of rapamycin), has also been suggested as a possible mediator of rapid antidepressive effects of ketamine and of long-term Flx treatment, with contrasting results [29,30].

Both adaptations to altered neurotransmission and remodelling of neuronal networks suggested as a substrate for the long-term effects of antidepressants involve the progressive adaptation of genetic regulation pathways. Multi-omics analysis of several brain regions of mice treated with Flx for six weeks have revealed a complex, region-specific genetic signature, which includes increased energy metabolism via oxidative phosphorylation and mitochondrial changes, chromatin remodelling and transcription factor regulations [31]. Large epidemiological studies demonstrate that MDD has a strong familial component, associated with heritable genetic influences that are modulated by patient-specific environmental exposures [32,33,34]. Similarly, the efficiency of SSRI treatment on depression is highly variable depending on the patient, suggesting an important role of the genetic background in both the susceptibility to depression and the response to the treatments [35].

In order to better understand the genetic mechanisms implicated in depression and its treatment, the effects of targeted genetic mutations on the responses to antidepressants were studied in the mouse. Some of these mutant mice, even in the absence of pharmacological treatment, showed an “anti-depressant-like” phenotype, with behavioural and histological signs reminiscent of those induced by the chronic administration of antidepressants, highlighting the role of the targeted genes for the mechanism of action of the drugs [19,36,37].

*Dlx* genes encode a family of homeodomain transcription factors involved in many developmental processes [38,39]. In mammals, six *Dlx* genes are arranged in three bigenic clusters [40]. In particular, *Dlx5* and *Dlx6* are expressed by developing and mature telencephalic and by a pool of diencephalic GABAergic neurons, but not in other brain regions [38,41,42]. *Dlx5* and *Dlx6* are co-regulated and present redundant functions [43].

In the cerebral cortex, Dlx5/6 are particularly important for the differentiation of PV-positive GABAergic interneurons [44]. Heterozygous *Dlx5/6* inactivation in the mouse results in abnormal prefrontal cortex gamma (γ; ~30–120 Hz) oscillations, which depend on PV-interneurons activity and in working memory deficits [45]. Targeted inactivation of *Dlx5/6* in mouse GABAergic neurons alters behaviour, vocal socialisation and metabolism with a reduction in anxiety-like and obsessive-compulsive-like behaviours [41,46]. Recently, we have shown that the density of PV-positive neurons in the adult prefrontal cortex and in the hippocampus is directly correlated with the *Dlx5/6* allelic dosage [47]. In particular, mice with reduced or absent *Dlx5/6* expression in GABAergic neurons present a reduced number of mature PV neurons in the frontal cortex and show reduced anxiety, a peculiar response in the “marble burying test” with some mice not burying any marble, which can be interpreted as a strong reduction in anxiety-like behaviours. Remarkably, the phenotypes obtained after *Dlx5/6* GABAergic inactivation are reminiscent of phenotypes of mice treated with fluoxetine or other SSRIs [18,48]. On the other hand, experimental overexpression of *Dlx5* in GABAergic neurons results in increased PV-neuron density in the frontal cortex associated with increased anxiety [47].

*DLX5* is imprinted in the human brain [49], and partially imprinted in the mouse with preferential transcription of the maternal allele. The genomic methylation regulator, MECP2, binds to the genomic region including *DLX5/6*; it is deregulated in Rett syndrome, an X-linked neurodevelopmental disorder that provokes a *DLX5/6* overexpression [50].

The *DLX5/6* genomic region also contains a long non-coding RNA (LncRNA), *DLX6-AS1* and *DLX6-AS2* (*Evf2* and *Evf1*), which form ribonucleoproteic complexes to regulate gene expression, including *DXL5/6* [51,52]. *DLX6-ASs* also bind to other non-coding RNAs to regulate their actions, for instance, *DLX6-AS1* indirectly regulates the BDNF pathway through its interaction with *miR-107* in neuroblastomas [53].

A large-scale transcriptomic analysis of post-mortem brains has shown that the *DLX5/6* locus participates in genetic modules altered in GABAergic neuronal function in autism spectrum disorders (ASDs) and schizophrenia [54]. Namely, *DLX6-AS1* has been identified as the most central hub gene in the interneuron module downregulated in schizophrenia and ASD. Patients carrying mutations in the *DLX5/6* brain-specific enhancers, which include the *I56i*, *I56ii* and *MEF2* [55,56], present a higher incidence of ASD and speech delay [11,57,58,59].

Mutations involving lifelong brain *Dlx5/6*-altered expression are, therefore, associated with cognitive modifications both in humans and mice.

In this study, we show that Flx administration induces a rapid and persistent reduction in *Dlx5* and *Dlx6* expression in the adult brain through the TrkB-CREB signalling cascade and induces phenotypic alterations reminiscent of *Dlx5/6* invalidation. These new findings identify the regulation of *DLX5/6* as a target to decipher the action of antidepressants.

## 2. Materials and Methods

### 2.1. Animals

Procedures involving animals were conducted in accordance with European and French Agriculture Ministry directives. The project was reviewed and approved by the MNHN “Cuvier” ethical committee and validated by the “Ministère de l’Enseignement Supérieur et de la Recherche” (Apafis 37771).

Mice were housed in light—(12 h cycle), temperature—(21 °C) and humidity—(50–60% relative humidity) controlled conditions. Food and water were available ad libitum.

Mice from the *Dlx5/6^flox/flox^* strain were bred with *Vgat^cre/+^* mice to obtain *Dlx5/6^VgatCre/+^*, *Dlx5/6^VgatCre^*, as previously described [46].

*R26R^CAG-flox-Dlx5/+^* mice were crossed with *Vgat^cre/+^* to induce GABAergic-specific expression of *Dlx5* (*GABAergic^Dlx5/+^* mice) as previously described [47]. In all experiments involving these mouse lines, littermates with no *Vgat^cre^* alleles were used as controls.

C57BL/6 (Charles River, Saint-Germain-Nuelles, France) male mice were used for pharmacological studies on wild-type animals.

In all experiments, 6 to 8 2-month-old mice were randomly attributed to experimental groups.

### 2.2. Animal Groups and Drug Administration

We followed two different protocols for Flx (Clinisciences, Nanterre, France) administration:

(a) For long-term chronic treatments, Flx was delivered ad libitum in drinking water at a dose of 160 mg/L, which has been shown to be equivalent to 18 mg/kg/day IP Flx injections resulting in an average Flx serum concentration of 500 ng/mL [60,61]. The drinking solution was changed every two days. All animals except *GABAergic^Dlx5/+^* mice were divided into four equivalent groups: “Vehicle” group received only water while treated groups received Flx in drinking water for the number of days indicated in each individual experiment (3D, 7D, 21D or 28D).  Similarly, *GABAergic^Dlx5/+^* mice and their control littermates “Control” were divided into three groups: the first one received only water while the two others were treated with Flx for 7 days or 3 weeks (7D, 3W).

(b) For short-term acute treatments, Flx was dissolved in 0.9% NaCl and administered intraperitoneally (15 mg/kg/day [60]) to C57/BL6 mice. Brains were collected 12 h or 24 h after the injection. The control group received only an IP saline injection (NaCl 0.9%) and was labelled as “Vehicle”.

For treatment with Flx combined with other drugs, mice were randomly divided into groups of equivalent size. All drugs were individually administered with a single intra-peritoneal injection in a volume of 0.1 mL/10 g of body weight. For co-treatments of Flx, ANA-12 (0.5 mg/kg in 1% DMSO [62], SML0209 Merck, Saint Quentin Fallavier, France), 666-15 (10 mg/kg in 10% DMSO and 90% Oil [63], 5383410001 Merck, Saint Quentin Fallavier, France) and rapamycin (10 mg/kg in 1% DMSO [64], 37094 Merck, Saint Quentin Fallavier, France) were individually administered 2 h before Flx (15 mg/kg); animals were dissected and analysed 24 h after Flx administration. 7,8-DHF (5 mg/kg in 1% DMSO [65], D5446 Merck, Saint Quentin Fallavier, France) was administered 24 h before dissection and analysis.

### 2.3. Reverse Transcription Quantitative PCR (RT-qPCR)

Cortical fragments from the somatosensory area were micro-dissected from 1 mm thick brain sections under a stereomicroscope in cold Tris buffer and immediately frozen in dry ice. Fragments were homogenised with a tissueLyser (QIAGEN, Courtaboeuf France) and total RNA was isolated using an RNAquerous-Micro Kit (Invitrogen, ref.: 1931, Courtaboeuf, France) according to the manufacturer’s instructions. On-column deoxyribonuclease digestion (Kit Turbo DNA free, ref.: AM1907, Appliedbiosystems, Villebon sur Yvette, France) was applied after RNA isolation to remove potential genomic DNA contamination. cDNA was synthesised from 1 µg of RNA (Invitrogen, ref: 10777-019, Courtaboeuf, France). Real-time PCR (RT-PCR) was performed using the SYBR Green method according to the manufacturer’s instructions (*Power* SYBR^TM^ Green PCR Master Mix, ref.: 4367659 Applied Biosystems, Villebon sur Yvette, France). The comparative Ct method on MxPro qPCR software (version 4.01, Agilent Technologies, Les Ulis, France) was used to determine the normalised changes in the target gene relative to *Actin B* and *β3-Tubulin* mRNA.

*Dlx5*, *Dlx6* and *Dlx6-AS1* transcripts were analysed using the primers listed in Table 1. Primers were tested and validated on previous experiments performed on *Dlx5/6*-invalidated mice [41].

When appropriate (Figure 3), qPCR analyses were performed on samples deriving from the same animal that had undergone a forced swim test.

### 2.4. Forced Swim Test (FST)

Mice behaviour was tested between 9 am and 4 pm.

Mice were taken to the dim and quiet test room 30 min before the test. A cylindrical transparent container (20 cm height × 14 cm internal diameter) was filled with 15 cm of warm water (29 °C). Mice were individually placed in the water and filmed for 6 min. The time during which the animals kept their heads above the water without actively swimming (immobility time) was measured manually with a chronometer in the last 4 min of recording. After completion, mice were removed from the tank, gently dried with a clean paper towel and placed in a clean, heated cage (37 °C).

### 2.5. Immunohistochemistry

Immunohistochemical analysis was performed on sections from the same mice that underwent behavioural tests. Animals were deeply anesthetised and perfused intracardially with 4% paraformaldehyde in phosphate buffer. Brains were removed and postfixed overnight at 4 °C in the same fixative and cryoprotected by immersion in 30% sucrose for at least 24 h. Cryoprotected brains were embedded in Tissue-tek (Leica cat.: 3801480, Nanterre, France) and 60-micron-thick free-floating cryostat sections of the whole brain were prepared. Sections including the prefrontal and parietal cortices were incubated in blocking solution (PBS 1%, 2% gelatine and 0.25% Triton) before overnight incubation with mouse anti-PV, 1:2000 (P3088, Merck, Saint Quentin Fallavier, France) at 4 °C.

Sections were then incubated with Alexa 480 Donkey anti-mouse (Jackson ImmunoResearch 715-545-151, Interchim, Montlucon, France) at room temperature for 2 h. After mounting in Fluoromount-G mounting medium (ThermoFisher, Villebon sur Yvette, France), brain sections were observed with a Leica DM6 B LED fluorescence microscope, photographed and specific cortical areas were delimited as in [47]. PV-positive neurons were counted manually with the “Fiji” programme counting tool and their density was calculated by dividing this number by the surface area. Deep and superficial regions were determined on cytoarchitectural criteria using DAPI counterstaining. This operation was repeated on the left and right hemi-cortices and the results were averaged. Quantifications were performed blind to genotype/treatments.

### 2.6. Statistical Analysis

The central line on box-and-whisker plots denotes the median, edges are upper/lower quartiles, whiskers show minimum/maximum values, points are individual experiments. Mann–Whitney (comparison of two groups), Kruskal–Wallis (comparison of more than 2 groups) and Friedman (repeated measures Figure 3B) tests were performed using Prism (Graphpad Software, version 10.2.3, La Jolla, CA, USA). Values of *p* < 0.05 were considered statistically significant.

## 3. Results

### 3.1. Dlx5/6 Expression in GABAergic Neurons Is Correlated with Depressive-like Phenotypes

**Figure 1:**
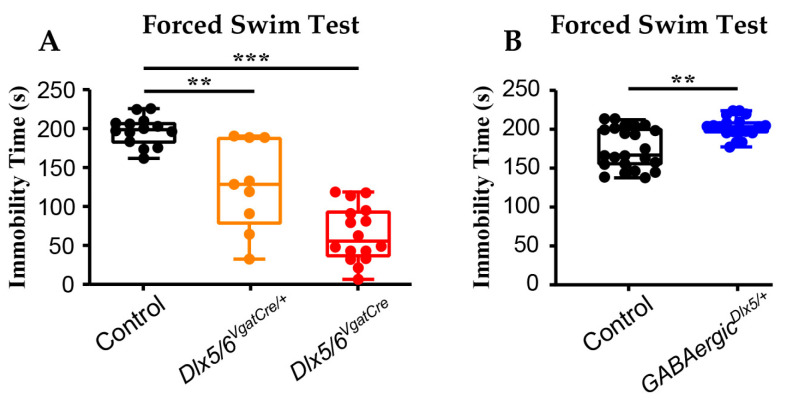
*Dlx5/6* expression in GABAergic neurons is correlated with immobility time in the forced swim test. Adult *Dlx5/6^VgatCre/+^* (*n* = 9), *Dlx5/6^VgatCre^* (*n* = 16) and control littermates (*n* = 14) (**A**) and *GABAergic^Dlx5/+^* and control littermates (*n* = 23) (**B**) mice and their corresponding control littermates were submitted to the forced swim test (FST). Mice with reduced (*Dlx5/6^VgatCre/+^*) or absent (*Dlx5/6^VgatCre^*) *Dlx5/6* expression in GABAergic neurons remained immobile, respectively, 35% and 70% less than their controls during the last 4 min of the test (**A**), whereas mice with an overexpression of *Dlx5* in GABAergic neurons (*GABAergic^Dlx5/+^*) spent 21% more time immobile than their own control littermates (**B**). Mann–Whitney and Kruskal–Wallis tests ** *p* < 0.01, *** *p* < 0.001.

We have previously shown that heterozygous (*Dlx5/6^VgatCre/+^* mice) and homozygous (*Dlx5/6^VgatCre^* mice) *Dlx5/6* invalidation in GABAergic neurons result in reduced anxiety-like behaviours and in a decreased density of prefrontal PV-neurons [41,47], while overexpression of *Dlx5* in GABAergic neurons (*GABAergic^Dlx5/+^* mice) [47] induces opposite phenotypes. Since both anxiety and prefrontal density of PV-interneurons are correlated with depression-like behaviours, we performed a forced swim test on *Dlx5/6^VgatCre/+^*, *Dlx5/6^VgatCre^* and *GABAergic^Dlx5/+^* mice to measure their depressive-like status. Although this test was designed for drug screening in acute treatments, it was also used to measure native depressive-like behaviours in non-treated mouse strains [66]. Reduction in *Dlx5/6* expression induced a 35% and a 70% reduction in the immobility time, respectively, in *Dlx5/6^VgatCre/+^* and *Dlx5/6^VgatCre^* mice compared to the controls (Figure 1A). In contrast, in *GABAergic^Dlx5/+^* mice, in which *Dlx5* is overexpressed in GABAergic neurons, we observed a 21% increase in the immobility time compared to the controls (Figure 1B). Together, these findings suggest a correlation between the cortical *Dlx5/6* expression levels and depressive-like phenotypes.

### 3.2. Fluoxetine Induces a Rapid Reduction in Cortical Dlx5/6 Expression

**Figure 2:**
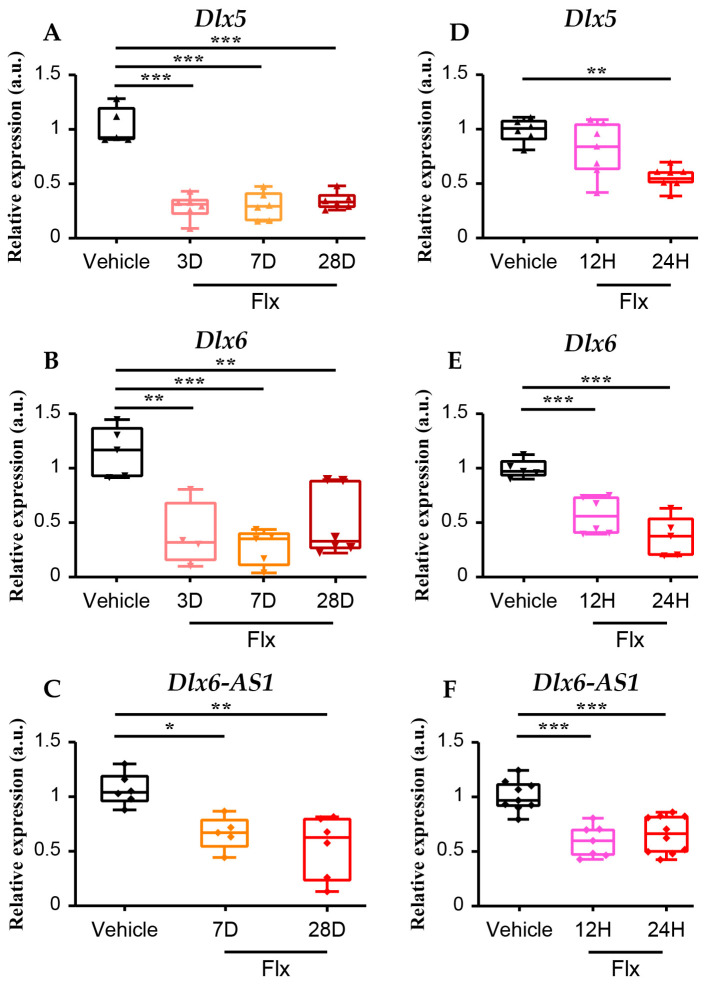
Fluoxetine administration induces a rapid and sustained inhibition of *Dlx5*, *Dlx6* and *Dlx6-AS1* expression in the cerebral cortex. C57BL/6 adult mice were treated with Flx or vehicle, and the effects on *Dlx5*, *Dlx6* and the LncRNA *Dlx6-AS1* cortical expression were measured by qPCR. (**A**–**C**) Chronic administration of fluoxetine in drinking water resulted in a stable 75% reduction in *Dlx5* and *Dlx6* expression after 3, 7 or 28 days of treatment (**A**,**B**) and a 50% reduction in the expression of *Dlx6-AS1* (**C**) (vehicle *n* = 5; Flx 3D *n* = 5; Flx 7D *n* = 5; Flx 28D *n* = 6). (**D**–**F**) Short-term effects of fluoxetine administration were tested after a single Flx IP injection. At a time of 12 h after the administration, the cortical expression of *Dlx6* was reduced by 47% (*n* = 6 per group) (**E**) and *Dlx6-AS1* by 38% (*n* = 7 per group) (**F**) whereas *Dlx5* expression levels were highly variable (*n* = 6 per group) (**D**). At a time of 24 h after the injection, we observed a reduction in *Dlx5* expression of 38% (*n* = 7 per group) (**D**), in *Dlx6* expression of 64% (*n* = 6 per group) (**E**) and in *Dlx6-AS1* expression of 40% (*n* = 10 per group) (**F**) in the cerebral cortex. Kruskal–Wallis test * *p* < 0.05, ** *p* < 0.01, *** *p* < 0.001.

In order to confirm the correlation between *Dlx5/6* expression and depressive-like status, we treated mice with fluoxetine, which is known to have an antidepressant action in 3 weeks of treatment in both humans and rodents. Fluoxetine (Flx) was administered chronically in drinking water (160 mg/L) to C57BL/6 male mice, and we measured *Dlx5/6* expression in the cerebral cortex by qPCR at different time points. We observed a 75% reduction in both *Dlx5* (Figure 2A) and *Dlx6* (Figure 2B) expression induced by fluoxetine at any stage tested from 3 to 28 days of treatment. The *Dlx5/6* locus also generates *Dlx6-AS1*, a long non-coding RNA, which forms complexes with nuclear proteins to regulate their action. After chronic Flx administration, the expression of *Dlx6-AS1* expression was also reduced by 40% 7D after the beginning of the treatment (Figure 2C). In order to better control the kinetics of Flx’s rapid effects, we administered Flx by intraperitoneal injection (15 mg/kg). We observed that cortical *Dlx6* expression was reduced by 47% as early as 12 h after Flx injection (Figure 2E), whereas at this time point, the level of *Dlx5* expression was highly variable between individuals and did not present a significant difference compared to the saline-injected animals (Figure 2D). A significant reduction in both *Dlx5* and *Dlx6* cortical expression levels was observed 24 h after Flx injection (38% and 63%, respectively) (Figure 2C,D). *Dlx6-AS1* expression was reduced as early as 12 h after Flx administration (38%) (Figure 2F).

These results indicate that Flx administration has a rapid and sustained inhibitory action on *Dlx5*, *Dlx6* and *Dlx6-AS1* expression in the cerebral cortex.

### 3.3. Dlx5 Overexpression in GABAergic Neurons Conteracts the Antidepressant Effects of Flx

**Figure 3:**
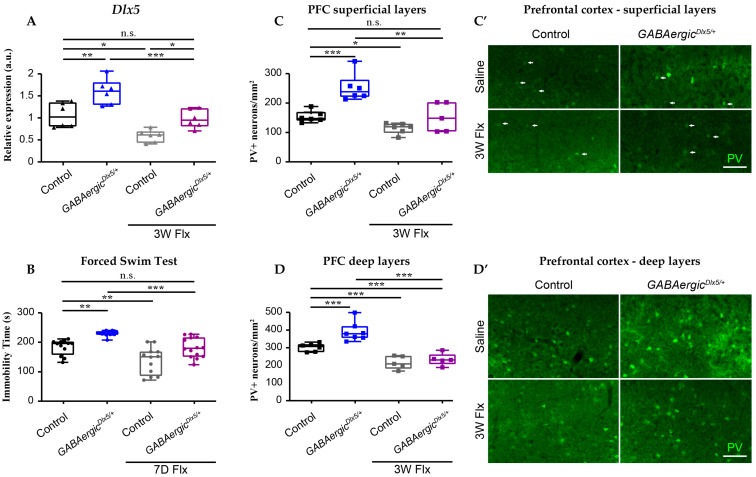
Fluoxetine administration counteracts the phenotypes induced by *Dlx5* overexpression in GABAergic neurons. (**A**) Control (*n* = 6) and *GABAergic^Dlx5/+^*mice (*n* = 7) were chronically treated with Flx or vehicle in drinking water for 3 weeks and the cortical expression of *Dlx5* was measured by qPCR. (**A**) Flx treatment induced a reduction in cortical *Dlx5* expression compared to vehicle-treated mice both in control and *GABAergic^Dlx5/+^* mice. After Flx treatment of *GABAergic^Dlx5/+^*mice, their cortical *Dlx5* expression became similar to that of vehicle-treated control animals. (**B**) The depressive-like behaviour of animals was analysed in the forced swim test. A chronic 7 days’ Flx treatment induced a decrease in immobility time both in control (*n* = 12) and *GABAergic^Dlx5/+^*mice (*n* = 14), resulting in an immobility time of Flx-treated *GABAergic^Dlx5/+^* comparable to vehicle-treated control animals, suggesting that Flx treatment had counteracted the depressive-like behaviour induced by *Dlx5* overexpression. (**C**–**D’**) The density of PV-positive neurons was measured in the prefrontal cortex of control (*n* = 7) and *GABAergic^Dlx5/+^* mice (*n* = 7) after a chronic 3 weeks’ Flx treatment (*n* = 6) compared to vehicle. PV-positive neuronal density was increased in the superficial (**C**,**C’**) and deep layers (**D**,**D’**) of the prefrontal cortex in *GABAergic^Dlx5/+^*mice compared to controls (arrows in (**C’**): PV-positive neurons). Flx treatment reduced PV+ neuronal in both genotypes, resulting in an equivalent PV+ neuronal density of the two Flx-treated groups. Friedman and Kruskal–Wallis tests n.s.: non-significant (*p* > 0.05), * *p* < 0.05, ** *p* < 0.01, *** *p* < 0.001, bar in (**C’**,**D’**) = 100 µm.

In order to test the hypothesis that the mechanism of Flx action involves the regulation of the *Dlx5/6* locus, we made use of our *GABAergic^Dlx5/+^* mouse line, in which *Dlx5* is overexpressed in GABAergic neurons by regulatory elements not present in the endogenous gene. These mice present a 55% increase in cortical *Dlx5* expression (Figure 3A) accompanied by an increased density of PV+ neurons in both the superficial and deep layers of the prefrontal cortex (Figure 3C,D). Treatment of this strain with Flx in drinking water (160 mg/L) for 3 weeks resulted in a 50% reduction in the level of cortical *Dlx5* expression, which became, therefore, similar to that of the saline-treated controls (Figure 3A). The immobility time in the forced swim test of *GABAergic^Dlx5/+^* was also reduced after a one-week-long Flx treatment and could not be distinguished from that of the control animals (Figure 3B).

After 3 weeks of Flx treatment, the density of PV+ neurons was reduced in the superficial and deep prefrontal cortical layers of the control and *GABAergic^Dlx5/+^* mice compared to the vehicle-treated littermates (Figure 3C,C’,D,D’). Together, these results suggest that (1) Flx silencing the endogenous *Dlx5/6* locus counterbalances the cellular and behavioural effects induced by *Dlx5* overexpression and (2) Flx administration does not have an antidepressive action if *Dlx5/6* expression is not reduced.

### 3.4. Dlx5 Expression Is Inhibited by the Activation of a TrkB-CREB Signalling Pathway

In order to analyse the intracellular pathway implicated in the rapid Flx-mediated *Dlx5/6* expression regulation, we tested the implication of the TrkB signalling pathway, which mediates the rapid effect of fluoxetine and other antidepressants. To avoid indirect effects that could appear after long times of exposure, we focused on the effects observed after 24 h of Flx treatment, the earliest time point presenting a significant and reproducible effect (Figure 2).

**Figure 4:**
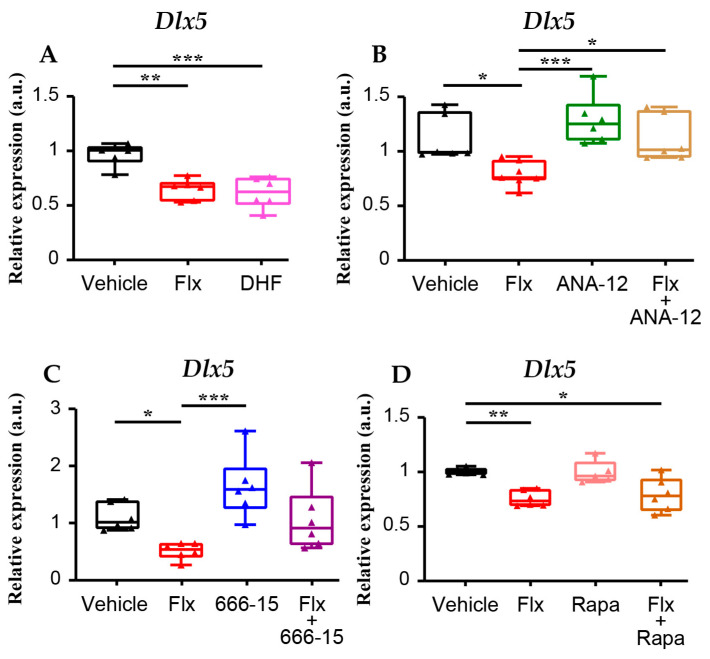
*Dlx5* expression is inhibited by the activation of a TrkB-CREB signalling pathway. C57BL/6 mice were treated with IP injections of Flx and of compounds interacting with the TrKB signalling pathways, and the effects on cortical *Dlx5* expression were measured after 24 h by qPCR. (**A**) 7–8DHF, a TrkB agonist, induced a reduction in *Dlx5* expression similar to that of Flx (vehicle *n* = 5, Flx *n* = 5; 7–8DHF *n* = 6). (**B**) The Flx-dependent inhibition of *Dlx5* expression was blocked in the presence of ANA-12, a TrkB antagonist (vehicle *n* = 6, Flx *n* = 7; ANA-12 *n* = 6, ANA-12 + Flx *n* = 6) (**C**,**D**) Flx was co-administered with antagonists of two TrkB major effectors: the CREB pathway, blocked by 666-15, and the mTor pathway, blocked by rapamycin. We observed that Flx’s action on *Dlx5* expression is blocked by 666-15 (*n* = 6 per group) (**C**) but is insensitive to rapamycin treatment (*n* = 6 per group) (**D**). Kruskal–Wallis test * *p* < 0.05, ** *p* < 0.01, *** *p* < 0.001.

Firstly, we compared Flx’s action with the effect of 7,8-dihydroxyflavone (7,8-DHF), a compound that is thought to be a TrkB receptor agonist, although recent research suggests that it may work through alternative mechanisms. We observed that they have a similar effect on *Dlx5* expression (Figure 4A). In contrast, the Flx-dependent regulation of *Dlx5* expression was blocked in the presence of ANA-12, a TrkB antagonist (Figure 4B).

In order to test the downstream TrkB effects, we co-administered fluoxetine with antagonists of two of its major effectors: the CREB pathway, blocked by 666-15 that inhibits CREB binding with its cofactor CREB-binding protein CBP, and the mTOR pathway, blocked by rapamycin. We observed that Flx’s action on *Dlx5* expression is blocked when CREB-mediated gene transcription is inhibited by 666-15 (Figure 4C) but is insensitive to rapamycin treatment (Figure 4D). Together, these results show that the TrkB-CREB signalling cascade mediates Flx’s fast action on *Dlx5* expression.

## 4. Discussion

*Dlx5* and *Dlx6* encode two homeodomain transcription factors expressed by GABAergic neurons and are involved in the development and postnatal maturation of Parvalbumin-positive fast-spiking cortical interneurons [44,47]. The starting point of this study has been the observation that a direct correlation exists between the levels of *Dlx5/6* expression in GABAergic neurons and the severity of depressive-like behaviours in the mouse: when *Dlx5/6* are deleted in these neurons, depressive-like behaviours are reduced whereas more severe signs of depression are observed when *Dlx5* expression is experimentally increased in the same neurons. Remarkably, treatment of normal mice with the antidepressant fluoxetine results in a rapid reduction in *Dlx5/6* expression and the same treatment can counteract the neuronal and behavioural effects of *Dlx5* overexpression. Most antidepressants, including Flx, increase the expression and signalling of brain-derived neurotrophic factor (BDNF) through neurotrophic tyrosine kinase receptor 2 (TrkB) [20,22], which activates a signalling cascade involving the transcription factor cyclic adenosine monophosphate response element-binding protein (CREB) [67]. TrkB stimulation, via agonists or Flx administration, inhibits *Dlx5/6* expression in GABAergic neurons through CREB signalling. Together, our findings suggest *Dlx5/6* as genetic effectors for the TrkB-dependent action of antidepressants.

### 4.1. A Genetic Regulation Underpinning Antidepressant Action in GABAergic Neurons

The important heritable component of MDDs [32,33,34] can be an entry point to understand the molecular and cellular origin of the disease and to design new diagnostic and therapeutic strategies. Large-scale GWAS studies [32], implicating hundreds of thousands of people, have revealed that depression susceptibility is highly polygenic with potential bias related to geographic origin. Many of the involved genes are implicated in the regulation of cerebral cortex activity, and also participate in genetic cascades implicated in other psychiatric diseases such as schizophrenia or autism spectrum disorders [68,69,70]. Single-nuclei RNA sequencing (snRNAseq) comparison of control and depressed cortical biopsies has permitted the differential analysis of the specific cell types that contribute to the disease [71]. A similar analysis aimed at detecting the mechanism of action of Flx revealed important differences in cell-specific gene expression in response to Flx treatment, but also significant variance between males and females [31,72]. These snRNAseq studies provide an important data reference to understand Flx’s mode of action. However, despite its power of analysis, a major limitation of scRNAseq analysis is its limited capacity to identify genes that present a low level of expression such as those transcription factors that, weakly expressed in adult tissues, are nonetheless essential for the maintenance of cellular function and differentiation. Since transcription factors regulate the expression of whole sets of genes, even modest variations in their expression can have a major impact on the cellular transcriptome. This is notably the case for *Dlx5* and *Dlx6* that are weakly expressed by all subtypes of adult cortical GABAergic neurons. Although their functions have been well studied during development, their role in adult Parvalbumin fast-spiking cortical GABAergic neurons is only partially understood [41,44,45,47]. Here, we have shown that the action of Flx, which is known to alter Parvalbumin neurons, is associated with a strong reduction in *Dlx5/6* expression. *Dlx5/6* inhibition likely occurs in most GABAergic interneurons and could alter the activity and function of cortical neuronal networks.

### 4.2. Flx Administration Affects Dlx5/6 Expression through the TrkB-CREB Signalling Pathway

We have shown that the reduction in *Dlx5* and *Dlx6* expression is detectable as early as 12 h, and highly significant 24 h after Flx or 7,8-DHF treatments.

This duration could correspond to a direct control of *Dlx5/6* expression by CREB signalling or be mediated by an intermediate transcription factor.

The delay between Flx administration and the reduction in *Dlx5/6* mRNA abundance in cortical extracts takes into account the kinetics of the different steps of the cascade including the bioavailability of the drugs, the signalling cascade to reach the transcriptional level and the stability of *Dlx5/6* mRNA.

Administration of antidepressants to a cAMP response reporter mouse line showed that the time required for CREB phosphorylation and CREB-dependent gene expression was greater than 6 h [73].

The stability of *Dlx5/6* mRNA is not known. However, analysis performed in human cell lines showed that mRNAs stability is very variable and can range from hours to days, with a median half-life of 10 h. Transcription factors are often “fast-decaying”, with half-lives that can be reduced down to less than 2 h [74].

These two elements strongly suggest that the CREB signalling cascade directly controls *Dlx5/6* expression. It has been suggested that 7,8-DHF may not be a BDNF agonist, instead binding with high affinity to other receptors [75]. We cannot exclude that 7,8-DHF or even Flx acts on *Dlx5/6* expression through other signalling pathways. However, the blockade of fluoxetine’s action on *Dlx5/6* expression by the 666-15 compound, which prevents the binding of CREB with its CBP cofactors, increases the likelihood of a direct fluoxetine–TrkB–CREB cascade in the regulation of *Dlx5/6* expression.

In support of this hypothesis is the observation that links between the CREB signalling cascade and *Dlx5/6* expression have been identified in other systems.

In mouse pre-adipocytes, it was shown that a CREB-C/EBP β complex inhibits *Dlx5* expression by binding a CREB-responsive element upstream of *Dlx5* [76]. Interestingly, the intergenic region of *Dlx5/6* includes another functional CREB binding site, which activation leads, in in vitro human and murine renal podocyte cultures, to an increased expression of the long non-coding RNA *DLX6-AS1* [77]. Strikingly, the location of the first *Dlx6-AS1* exons correspond to *Dlx5/6* intergenic promoters of central nervous system *Dlx5/6* expression, which could explain the discrepancies between the neuronal inhibition and renal stimulation of *Dlx5/6* expression in response to CREB signalling [77,78]. Similarly, with what we describe in the cerebral cortex, inhibiting CREB transcriptional function with the 666-15 compound prevents CREB action on *Dlx6-AS1* expression although the nature of the CBP could differ from one organ to another [77].

### 4.3. An Implication of DLX5/6 Gene Expression in Depression and Antidepressant Mechanisms

*Dlx5/6* are expressed by most forebrain GABAergic neurons and are involved in the development, differentiation and maintenance of PV-positive cortical neurons. Modifications of *Dlx5/6* expression targeted to mouse GABAergic neurons have permitted an association of anxiety-like, compulsive-like and social behaviours with the allelic dosage of these genes [41,46,47]. Here, we have shown that higher levels of *Dlx5/6* expression in GABAergic neurons are also associated with the exacerbation of depressive-like behaviours.

**Figure 5:**
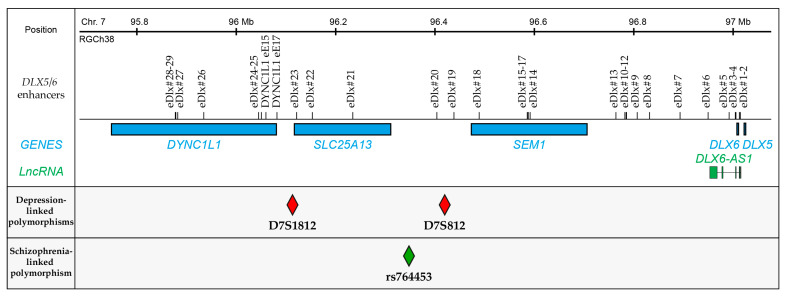
Human *DLX5/6* genomic region has long-range enhancers that include loci associated with depression and schizophrenia. The regulatory region governing *DLX5*, *DLX6* and *DLX6-AS1* expression spans over more than 1 MB 5′ from *DLX5* and contains at least 31 enhancers that control different aspects of genes and LncRNA expression from the locus. This region also contains 3 genes: *DYNC1L1*, *SLC25A13* and *SEM1*; some of the *DLX5/6/6-AS1* enhancers are located within their coding sequences. Genetic associations have identified two loci positioned on Chr7 by red diamonds: D7S1812 and D7S812, distant from *DLX5/6/6-AS1* but within their regulatory sequences, associated with depressive disorders in patients [79,81]. In South Asian populations, the genetic variant, rs764453, was associated with schizophrenia (green diamond) [80].

In humans, *DLX5/6* expression is under the control of regulatory sequences spanning over 1 Mb on chromosome 7 (7q21.3); this region includes several enhancers that have been linked to somatic and/or mental disorders, including cognitive and social defects [46]. Many studies have tried to better understand the genetic contribution to depressive disorders, which could both better explain familial susceptibility to developing depression or resistance to treatments. Genetic linkage experiments have identified the locus D7S821, recessively associated with bipolar disorders, as also being associated with major depressive disorders [79]. D7S821 is located in an intergenic region between the two identified *DLX5/6* enhancers *eDlx#19* (15 kb upstream) and *eDlx#20* (18 kb downstream) (Figure 5). D7S821 polymorphism linked to depression could therefore involve alterations in *DLX5/6* locus expression. Interestingly, a genetic variant, rs764453, located in the same intergenic region, was reported as being associated with schizophrenia in South Asian populations [80]. This variant is positioned 56 kb upstream from *eDlx#20*, 71 kb upstream from D7S821 (Figure 5). The association between rs764453 and schizophrenia is not found in Caucasian or Chinese populations. This difference likely comes from the different linkage disequilibrium of this SNP, which differs between geographic regions. These associations suggest a role of the polymorphism of the genomic region around rs764453 rather than the variant itself.

Patients with polymorphisms in another neighbour region, at the D7S1812 locus within the *SLC25A13* gene, often present epilepsy, schizophrenia or depression symptoms [81]. This could be linked to alterations in SLC25A13 associated with *DLX5/6* expression modifications since D7S1812 is located 8 kb upstream and 39 kb downstream from the *DLX5/6* enhancers *eDlx#23* and DYNC1l1 eExon 17, respectively (Figure 5). Deletions in the regulatory sequences of *DLX5/6* often result in a complex syndrome that include craniofacial defects and split hand–foot malformations due to altered *DLX5/6* developmental expression in the limbs; it can be associated with episodes of depression [82,83]. Interestingly, a patient was reported with a heterozygous 3 MB deletion upstream on the *DLX5/6* locus, including both the D7S812 and D7S1812 locus, with no alteration in the *DLX5/6/6-AS1* coding sequences. This person did not present with ectrodactyly malformation nor intellectual disability. However, he suffered from a paranoid personality associated with frequent depressive episodes [82,83]. Notably, the father of the patient reported in [82,83] was diagnosed with paranoid schizophrenia, and their extended family presented a high incidence of psychosis.

Direct alterations in *DLX5/6* expression are difficult to detect via single-cell RNAseq due to the low expression levels of these genes. However, the study of regulons, which correlates the expression of transcription factors with their targets, confirms a central role of *DLX5*, *DLX6* and the long non-coding RNA *DLX6-AS1* in the control of GABAergic cortical neuron functions. Alterations in regulons involving *DLX5/6* or *DLX6-AS1* have been implicated in psychiatric disorders such as autism and schizophrenia [54,84,85], as well as Alzheimer’s disease and major depressive disorders [86].

Interestingly, *DLX6-AS1* recruits protein partners, including transcription factors such as DLX2 [87], and the genomic methylation regulator MECP2 to control the expression of GABAergic neuron genes such as *DLX5*, *DLX6* and *GAD67* [87,88]. MECP2 is a repressor of *DLX5/6* expression through the methylation of the locus [50]. The phosphorylation of MECP2 has been proposed as a mediator for the long-term effect of fluoxetine and fast-acting antidepressants, which could therefore provoke a sustained repression of the *DLX5/6* locus [25,89]. The level of *Dlx5/6* locus methylation is also controlled by demethylases, such as Gadd45b, which also targets *Gad1* and *Ntrk2* (TrkB) regulatory sequences, with an impact on these genes’ expression levels [90]. Elucidating the regulation of these genes might be a key to understanding the mechanism of action of antidepressants.

Despite decades of research, many aspects of depressive disorders and antidepressant action still remain to be better understood. In particular, there is missing knowledge about how pharmacological treatments translate into the cellular adaptations that lead to a behavioural phenotype. Although increasing evidence implicates the BDNF-TrkB-CREB pathway in this process, the downstream effectors remain elusive.

Our results suggest that the transcription factors DLX5/6 and the long non-coding RNA *DLX6-AS1* participate in this process. The role of these genes as transcriptional regulators positions them as orchestrators of GABAergic neuronal function as has been shown by post-mortem snRNAseq analysis on the brain of schizophrenic patients.

## 5. Conclusions

*Dlx* genes have been shown for a long time to be necessary for the proper development of GABAergic neurons during embryogenesis [91]. Restriction of *Dlx5/6* expression alteration to the brain has permitted overcoming embryonic lethality and describes postnatal behavioural and neuronal defects in the adult [41,45,46,47,92]. In these experiments, alterations in *Dlx5/6* expression were present since the embryonic stages and lasted throughout life, with increased or reduced anxiety- and depressive-like phenotypes depending on their genotypes.

In the present manuscript, we showed that administration of Flx in the adult induces a rapid, efficient and sustained inhibition of *Dlx5/6* expression. The effects of Flx on the forced swim test and on the alteration in cortical Parvalbumin density is blocked in *GABAergic^Dlx5/+^* mice with *Dlx5* overexpression. The expression of *Dlx5/6/6-AS1*, which persists in the adult [41], may therefore be regulated throughout life to mediate cellular and behavioural adaptations.

The present findings suggest that the expression levels of *DLX5/6* and *DLX6-AS1*, regulating differentiation and function of Parvalbumin-positive neurons in the adult brain, also affects anxiety and depression and pave the way for further analyses on the mechanism of action of antidepressants.

## Figures and Tables

**Table 1:** Primers used in this study for RT-qPCR.

*Dlx5*	Fw: 5′ TCT CTA GGA CTGACG CAA ACA 3′ Rv: 5′ GTT ACA CGC CAT AGG GTC GC 3′
*Dlx6*	Fw: 5′ GCA GAC TCA ATA CCT GGC CC 3′ Rv: 5′ GTG TGG GTT ACT ACC CTG CT 3′
*Dlx6-AS1*	Fw: 5′ CTC CCT CCG CTC AGT ATA GAT TTC 3′ Rv: 5′ CCT CCC CGG TGA ATA TCT CTT 3′
*Actin B*	Fw: 5′ CAT TGC TGA CAG GAT GCAGAAGG 3′ Rv: 5′ TGC TGG AAG GTG GAC AGT GAG G 3′
*β3-Tubulin*	Fw: 5′ CAT CAG CGA TGA GCA CGG CAT A 3′ Rv: 5′ GGT TCC AAG TCC ACC AGA ATG G 3′

## Data Availability

The data underlying this article are available on reasonable request from the corresponding author.
